# The Potential Connectivity of Waterhole Networks and the Effectiveness of a Protected Area under Various Drought Scenarios

**DOI:** 10.1371/journal.pone.0095049

**Published:** 2014-05-15

**Authors:** Georgina O’Farrill, Kim Gauthier Schampaert, Bronwyn Rayfield, Örjan Bodin, Sophie Calmé, Raja Sengupta, Andrew Gonzalez

**Affiliations:** 1 Ecology and Evolutionary Biology Department, University of Toronto, Toronto, Ontario, Canada; 2 Biology Department, McGill University, Montreal, Quebec, Canada; 3 Département de géomatique (KGS), Département de biologie (SC), Université de Sherbrooke, Sherbrooke, Québec, Canada; 4 Stockholm Resilience Centre, Stockholm University, Stockholm, Sweden; 5 Departamento de conservación de la biodiversidad, El Colegio de la Frontera Sur, Chetumal, Quintana Roo, Mexico; 6 Geography Department, McGill University, Montreal, Quebec, Canada; Institut Pluridisciplinaire Hubert Curien, France

## Abstract

Landscape connectivity is considered a priority for ecosystem conservation because it may mitigate the synergistic effects of climate change and habitat loss. Climate change predictions suggest changes in precipitation regimes, which will affect the availability of water resources, with potential consequences for landscape connectivity. The Greater Calakmul Region of the Yucatan Peninsula (Mexico) has experienced a 16% decrease in precipitation over the last 50 years, which we hypothesise has affected water resource connectivity. We used a network model of connectivity, for three large endangered species (Baird’s tapir, white-lipped peccary and jaguar), to assess the effect of drought on waterhole availability and connectivity in a forested landscape inside and adjacent to the Calakmul Biosphere Reserve. We used reported travel distances and home ranges for our species to establish movement distances in our model. Specifically, we compared the effects of 10 drought scenarios on the number of waterholes (nodes) and the subsequent changes in network structure and node importance. Our analysis revealed that drought dramatically influenced spatial structure and potential connectivity of the network. Our results show that waterhole connectivity and suitable habitat (area surrounding waterholes) is lost faster inside than outside the reserve for all three study species, an outcome that may drive them outside the reserve boundaries. These results emphasize the need to assess how the variability in the availability of seasonal water resource may affect the viability of animal populations under current climate change inside and outside protected areas.

## Introduction

The synergistic effects of land use change, habitat loss and climate change are expected to affect species persistence by altering the distribution and connectivity of resources and habitat. These effects will have significant consequences for biodiversity conservation and management [1], [2]. Landscape connectivity allows species movement and dispersal, influencing the distribution of genes, resources and populations of many species [3], [4]. Landscape connectivity analysis encompasses both the ease with which an animal can move from one resource patch to another (the animal perspective, [5]), and the location and quality of resources (the landscape perspective) that will determine the species’ motivation to move [6], [7].

Changes in temperature and precipitation regimes predicted by climate change models [8] are likely to influence resource availability through changes in their abundance (e.g. fruits, water). Resource connectivity analyses are particularly important when resources found within habitat patches vary in space and over short (within years) and long (between years) time scales (e.g. [9]). Hence highly variable fluctuations in temporal resource availability make connectivity within a resource network dynamic and stochastic in space and time [10], which is expected to influence species movement patterns [11].

Resource connectivity studies should incorporate the temporal variability in the availability and connectivity of resources given the current predictions of climate change worldwide, the species’ differential use and accessibility to resources, and the amount of suitable habitat remaining after landscape connectivity is lost [12]–[14]. This is particularly relevant when areas with low or declining connectivity may not be able to support viable populations of some species over long periods of time [15]. Given current observations on the long-term effects of climate change, longer-term fluctuations in precipitation than the ones presented currently may affect water networks resulting in multi-annual trends in network connectivity [10], [11].

In the Yucatan Peninsula of Mexico, climate change is causing an increase in drought periods [16], which seems to be influencing the availability of resources and the movement patterns of animals (e.g. [17]). The Greater Calakmul Region of the Yucatan Peninsula of Mexico is a continuous forested area in a highly seasonal tropical climate where, over the last 50 years, annual precipitation decreased by 16% while drought frequency increased [16]. Yearly fluctuations in precipitation show a decreasing pattern despite reports of years with high precipitation (Comisión Nacional del Agua, unpublished data). According to regional climate models, this area will increasingly suffer from extreme droughts in future years [8]. This area is a karstic upland area, where freshwater is only available to wildlife and people in superficial waterholes and small seasonal streams. Therefore, water is a scarce and dynamic resource in the area. Many small waterholes dry up during the dry season, and if the reduction in precipitation continues, we hypothesize this will further influence the availability of waterholes. In addition, we hypothesize that if drought events affect surface water availability (i.e., waterhole presence), waterhole connectivity will decrease given that waterholes that remain in the landscape will be located beyond species maximum travel capabilities or home ranges.

We used a network (graph theoretical) analysis to test our hypotheses about the change in the spatial distribution of waterholes in this network [18]. We treated these waterholes and short seasonal streams as nodes, and we defined a link between any two if they fell within the range of our study species’ movement distances [19], [20]. We modelled the movement of Baird’s tapir (*Tapirus bairdii* Gill, 1865), white-lipped peccary (*Tayassu pecari* Link, 1795) and jaguar (*Panthera onca* Linnaeus, 1758). We used these species because they are of significant conservation concern, they rely on waterholes, and data about their movement are available. We assessed the connectivity of waterholes for each species given the temporal and spatial changes of water availability within and between years inside and outside the Calakmul Biosphere Reserve considering actual observations and climate change predictions of severe droughts for the area. We used reported travel distances for our study species, rather than measuring the actual movement of individuals; our results therefore represent the potential connectivity network of waterholes. Although other studies have evaluated the importance of seasonal water resources for species survival [11], [12], [21], none have addressed the connectivity of water resource under scenarios of climate change and how resource availability interacts with species movement capacities to modify the functional connectivity of a landscape.

## Materials and Methods

### Study Area

**Figure 1:**
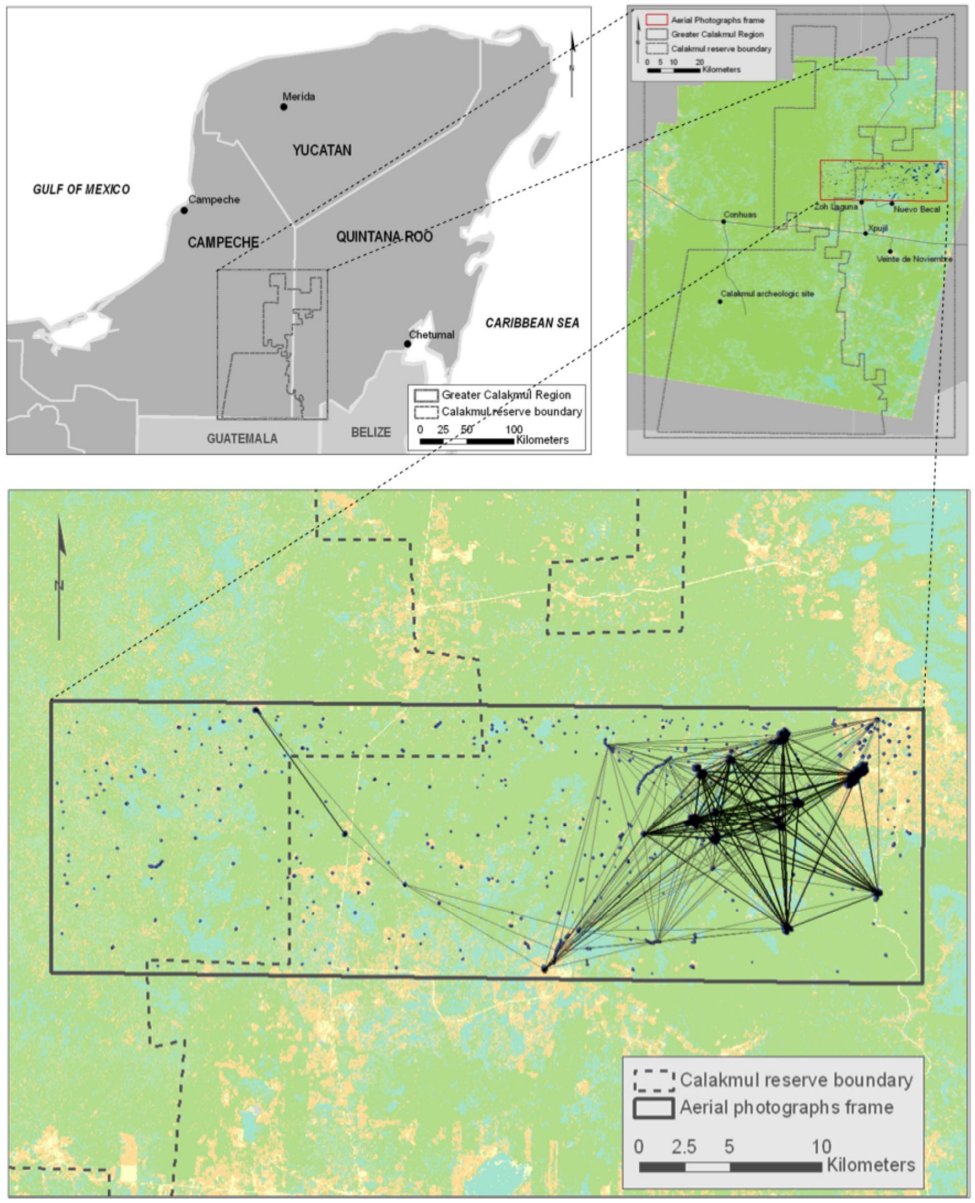
Greater Calakmul Region and study area (upper figures). This figure shows a representation of drought scenario D (grey links) and E (black links) for the 13 km travel distance. Links are lost inside and adjacent to the reserve and a narrow stepping-stone strip of waterholes remains, connecting the interior of the reserve with the smaller sub-network to the east, outside the reserve. The circle shows the waterhole that maintains the connectivity between the sub-networks.

The waterhole network is situated in an area of approximately 750 km^2^ located in the north-eastern part of the Greater Calakmul Region to the south of the Yucatan Peninsula of Mexico (19°15′ to 17°50′N and 90°20′ to 89°00′W). Fieldwork activities inside and in the areas surrounding the Calakmul Biosphere Reserve were authorized by the Director of the Reserve (Fernando Durand Siller- Permit no. D-RBC-020-10-07). Fieldwork outside the reserve was carried out in the Ejido of Nuevo Becal (communal land) and the Community commissioner authorized field activities after the assembly of the community was notified and agreed on allowing our visit to their land. Even though this study considers movement capacities of endangered species, data were collected from the literature and field activities did not involve any of the endangered species studied. The Greater Calakmul Region (Fig. 1) is part of the Selva Maya, the second largest area of tropical forests in the Americas. Approximately 13% of the forest cover is disturbed by human activities in the region [22]. Of our study area, 30–40% lies within the Calakmul Biosphere Reserve, while the eastern section represents a continuous forested landscape that corresponds to communal land and is mostly a managed forest reserve. Forest cover within and outside the Calakmul Biosphere Reserve does not show any large difference [22]; however, there are human settlements outside the borders of the reserve, which represent a threat to species, i.e. hunting and logging activities.

Between 1953 and 1998 precipitation decreased by almost 16% in the Calakmul Region; mean precipitation declined from 1,300 mm in 1950s to 790 mm in 1990s [23] following the same pattern until 2005 (1955–2005; Comisión Nacional del Agua, unpublished data; [23]). In addition, climate models predict a further decline in precipitation and warmer temperatures, and suggest that most severe droughts in Mexico would occur during el Niño years affecting 50% of the area covered by deciduous tropical forest [8]. Due to its geomorphologic conditions (karst topography), the region does not have any rivers and the majority of the superficial water is stored in small depressions on the landscape: waterholes (locally called “*aguadas*”) and small seasonal streams [24]. No waterholes are formed by underground water in this area. Therefore, during the dry season these superficial water bodies represent the only available water source for many animal species.

### Study Species

In the Greater Calakmul Region, species such as the Baird’s tapir, white-lipped peccary and jaguar are endangered [25] [26] [27]. These species are highly associated with water bodies for water consumption, to regulate their body temperature (tapirs and peccaries [28]) or to find their prey (jaguars [27]). Given that water is only present in waterholes and short streams, species such as these depend on seasonal water bodies and the associated habitat surrounding them [27], [29]–[31].

The number of water bodies throughout the landscape used by each species depends on their home ranges and daily movements. For individuals of Baird’s tapir, reported yearly mean home ranges encompass approximately 1.3 km^2^ (±0.73 km^2^, SD) with a maximum home range of 2.3 km^2^ in Costa Rica [32]. These authors reported that even though home ranges did not vary between seasons, during the wet season individual tapirs shared 26% of their annual home range with other tapirs, whereas overlap was usually null in the dry season; these observations suggest that water availability influences tapir use of the landscape. For white lipped peccaries, Reyna-Hurtado [30] estimated herd annual home ranges from 43.9 to 97.5 km^2^ in the tropical semi-dry forest of the Calakmul Biosphere Reserve (home range estimates based on VHF data and 95% fixed kernel). White-lipped peccaries visit waterholes disproportionately more often during the dry season than in the wet season [17]. During this study, when water became scarce and was only available at the larger waterhole in the area, the groups remained at this waterhole and foraged in a radius of 6 km (mean distance <600 m). In the Calakmul Region, jaguars show differential habitat use by season based on the availability of water bodies, which affects the density and location of prey species [27]. Data obtained from satellite collars showed that the activity area for two males was about 1000 km^2^ [27].

In addition to home ranges, maximum travel capacities provide information about the ability of a species to reach distant water bodies if required. In Peru, Tobler [33] reported lowland tapir (*Tapirus terrestris*) individuals moving up to 13 km over a 24-hour period (GPS radio-collared data), with a mean movement distance in a 24-hr period of 5.2 km (range 3.6–6.7 km). This is the only formal study documenting tapir movements with GPS collars. When considering tapir movements, we used the travel distances reported for this species of tapir; however, we are confident these observations can be used for the Baird’s tapir in our region as all tapir species depend on water for their survival [25]. Reyna-Hurtado *et al.* [17] found that white-lipped peccaries require visits to water bodies on an almost daily basis in our study region, performing search patterns at two spatial scales: they search one area intensively by moving no more than 3 km every day and occasionally perform long displacements (9 to >16 km) in a single direction that take them out of the previous searched area over the course of one to three days. For jaguars, the mean daily travelled distance was 2.24 km with a maximal daily distance travelled of 10 km based on radio collar data [34].

In summary our model species have been reported to move distances between 3 and 13 km (minimum and maximum reported travel distances by tapirs), between 3 and 16 km (minimum and maximum travel distances by white-lipped peccaries) and between 2.24 and 10 km (minimum and maximum travel distances by jaguars). This range (3–16 km) provides the minimum and maximum potential movement capacities of these species. This suggests that these species have the ability to reach waterholes located further away than their daily home ranges.

### Remotely Sensed Image Interpretation and Ground Truthing

For our analysis, we used remotely sensed imagery (orthophotographs from March 1998 and 2001) to obtain the most accurate locations of the waterholes in our study area (see File S1). During fieldwork, we observed that most of the waterholes smaller than 400 m^2^ were dry at the beginning of the dry season, therefore we only digitized water bodies >400 m^2^. We visited 15 waterholes observed in the orthophotos, confirming the presence of 80% of them in the field. All misidentified waterholes were small (<700 m^2^) and were subsequently excluded from the model. From 2006 to 2009, we repeatedly visited 15 waterholes and observed that large waterholes did not dry or were dry only during the peak of the dry season of very dry years. For example, in May 2006 a waterhole of 90,000 m^2^ was observed to go dry for the first time since people re-colonized the area 40 years earlier (Nicolas Arias, comm. pers). These observations support climate models that suggest an increase in drought conditions in the area and capture the situation during a very dry year (2005) in the region [8]. Animal tracks (e.g., tapir, deer, peccaries) and dung near small and large waterholes are found at lower densities when waterholes are dry, suggesting that animals use waterholes mainly for water [35]. The intervening matrix surrounding waterholes was comprised of accessible forest for all species; no major physical barriers (e.g. roads, mountains, rivers) occur in the study area.

### Waterhole Networks

Water resource connectivity was assessed for each of the three model species using species-specific waterhole networks. Various drought scenarios were simulated on these networks through node deletion sequences that reflected waterhole-drying patterns. Deletion sequences were based on observed waterhole drying during the dry season of 4 consecutive years (2006–2009). The implications of these drought scenarios on species-specific water resource connectivity were assessed by quantifying the structure of sub-networks, network-wide probability of connectivity, and access to waterhole-associated habitat. Our analysis thus provides an indication of potential future changes in waterhole connectivity based on current waterhole distribution and drying pattern.

### Waterhole Network Delineation

The three species’ networks consisted of the same set of nodes (waterholes), but each had a unique set of links reflecting the range of reported movement abilities of the three species, varying from 3 to 16 km (tapirs: 3–13 km; white-lipped peccaries: 3–16 km; and jaguars: 2.24–10 km). We identified links between waterhole centroids rather than waterhole edges due to high variability in waterhole edges with rainfall. Links that were longer than the maximum movement ability of each species were removed from their respective networks. Although we do not expect animals to necessarily travel their maximal distances in a day or constantly, these distances represent a reasonable estimate of the distance that these species might be able to travel to find water when it becomes limiting, e.g. to locate a new waterhole. Furthermore, in contrast to most resource networks where nodes represent patches of suitable habitat surrounded by an inhospitable matrix, in our study, nodes (waterholes) are separated by suitable habitat as the matrix landscape represents a continuous forested area. All waterhole networks were delineated using complete graph extraction in SELES (A Spatially Explicit Landscape Even Simulator; [36]).

### Waterhole Network Deletion Sequences Based on Drought Scenarios

The impact of drought on the connectivity of the waterhole network was modelled by removing nodes in increasing order of waterhole area. We made this assumption based on field observations that showed the largest waterholes were the last to become dry at the peak of the dry season from 2006 to 2009. We created a base scenario (A) that included all waterholes larger than 700 m^2^, and 10 drought scenarios (B–K) excluding waterholes smaller than or equal to (B) 1,000 m^2^, (C) 2,500 m^2^, (D) 5,625 m^2^; (E) 10,000 m^2^, (F) 15,625 m^2^, (G) 22,500 m^2^, (H) 30,600 m^2^, (I) 40,000 m^2^, (J) 50,600 m^2^ and (K) 62,500 m^2^. In the field, we measured the perimeter to evaluate the area of several waterholes that were monitored for water availability over the four years. The distribution of waterhole sizes allowed us to select our size class scenarios by providing maximum and minimum size limits. After deleting nodes in each scenario, only links shorter than the species-specific maximum movement distance were maintained within the species’ waterhole network. These pruned waterhole networks were used in the calculation of connectivity for each drought scenario.

### Waterhole Network Connectivity Analyses

Prior to modelling drought scenarios, we analysed the sub-network structure of the species-specific waterhole networks by enumerating the connected components (clusters). Network clusters represent connected areas of a network within which individuals can move among nodes (waterholes) via direct links or indirect paths [20], [37]. Different clusters are effectively isolated from each other as no links or paths allow individuals to move between them.

After applying each drought scenario, we recorded the total area covered by waterholes, the number of waterholes, and the density of links. We calculated link density (L) as the proportion of actual links present out of the total number of links in the equivalent complete network (L≤1). To evaluate the overall connectivity of the network we calculated the probability of connectivity defined as the probability that two individuals located randomly within the landscape are found at waterholes that belong to the same component [38]. Given the low proportion of water-covered area with respect to the total study area, we only considered the numerator of the probability of connectivity calculations to allow for better comparisons of results [38]. We derived a link attribute for dispersal probability by applying a negative exponential dispersal kernel to link lengths (m). We assumed a probability of 0.05 that species could move further than their maximum movement distance (straight line) to parameterize the negative exponential dispersal kernel [20]. We used the patch removal technique [39] to assess the contribution of each node in maintaining network connectivity for each species and under each drought scenario, which allowed us to rank waterholes based on their node importance to the network. This was measured as delta probability of connectivity [38], [40]. We calculated the area covered by the network or sub-networks using ArcGIS, and analysed network connectivity using the igraph package in R (R Development Core Team, 2005).

### Waterhole-associated Habitat

Our model species are strongly associated with water bodies in the study area. Therefore, we assumed that their habitat must contain at least one waterhole and defined the suitable habitat as the area surrounding that waterhole. We assume that species are in general unwilling to travel long distances in search for resources on a daily basis, thus their movement patterns will likely occur mostly within a small area surrounding a waterhole as has been observed for the white-lipped peccary [17]. Therefore, to evaluate the species suitable habitat, we created 1 and 2 km radius buffers around the edge of each waterhole in each scenario using ArcGIS 9.3 Spatial Analyst Tool (ESRI 2009). By considering these radii, we assume species will move short distances during the day <3 km (minimum daily travel distance reported by all species) around a waterhole if all their requirements are fulfilled in that area. This analysis allowed us to estimate the loss of suitable habitat for the species and to complement the network connectivity analysis where we focus on the ability for the species to undertake long and rare movements. In addition, we quantified the percentage of total area covered by the suitable habitat in each scenario.

## Results

**Figure 2:**
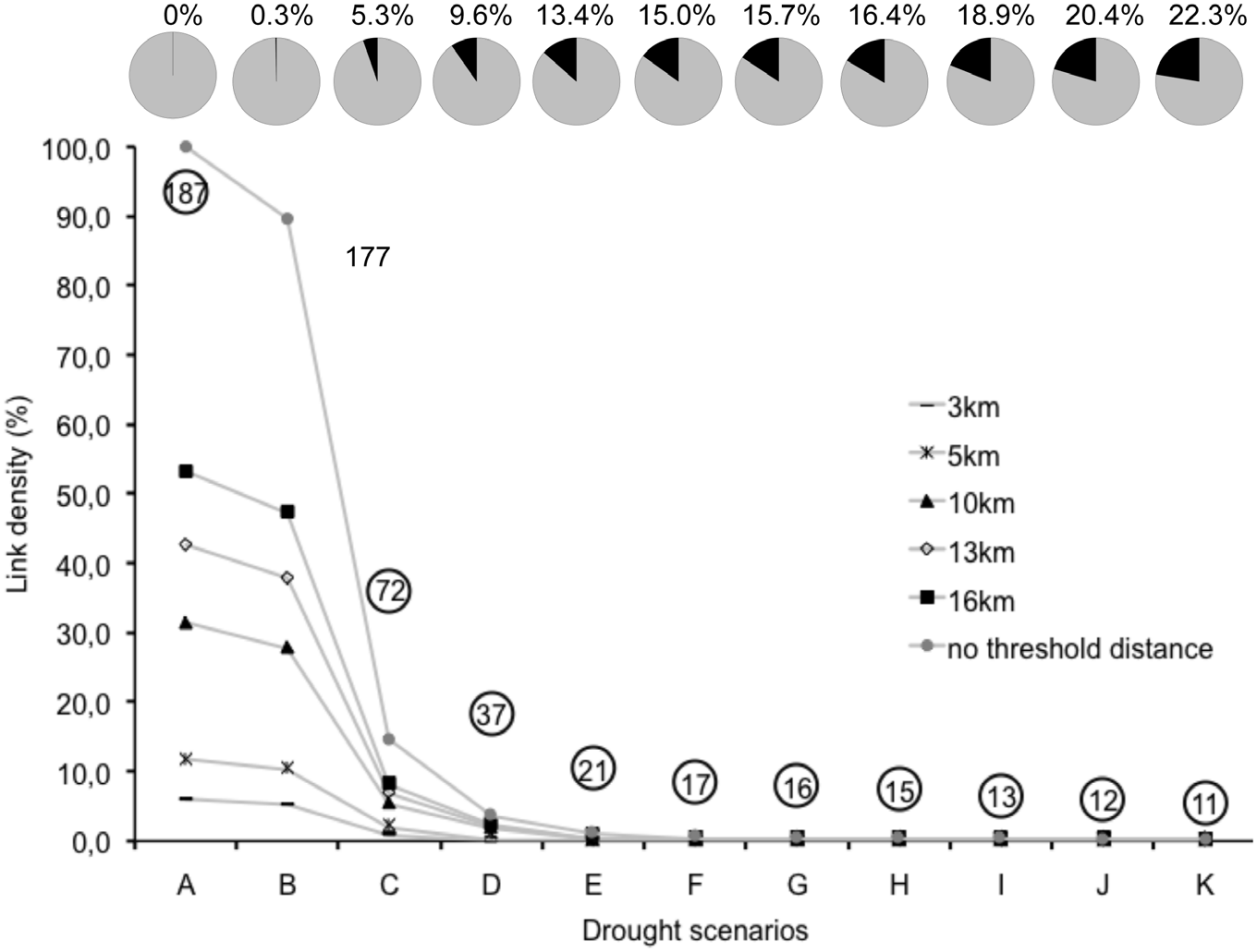
Number of waterholes (embedded in circle) and link density in each drought scenario by travel distances of species: tapirs (3–13 km), white-lipped peccaries (3–16 km) and jaguars (3–10 km). Drought scenarios correspond to waterhole removal based on waterhole size: A (all), B (≤1000 m^2^), C (≤2500 m^2^), D (≤5625 m^2^), E (≤10000 m^2^), F (≤15625 m^2^), G (≤22500 m^2^), H (≤30600 m^2^), I (≤40000 m^2^), J (≤50600 m^2^), K (≤62500 m^2^). Pie charts show the amount of waterhole area lost in each drought scenario. Lines between points are included only as reference to observe point-decreasing pattern.

**Figure 3:**
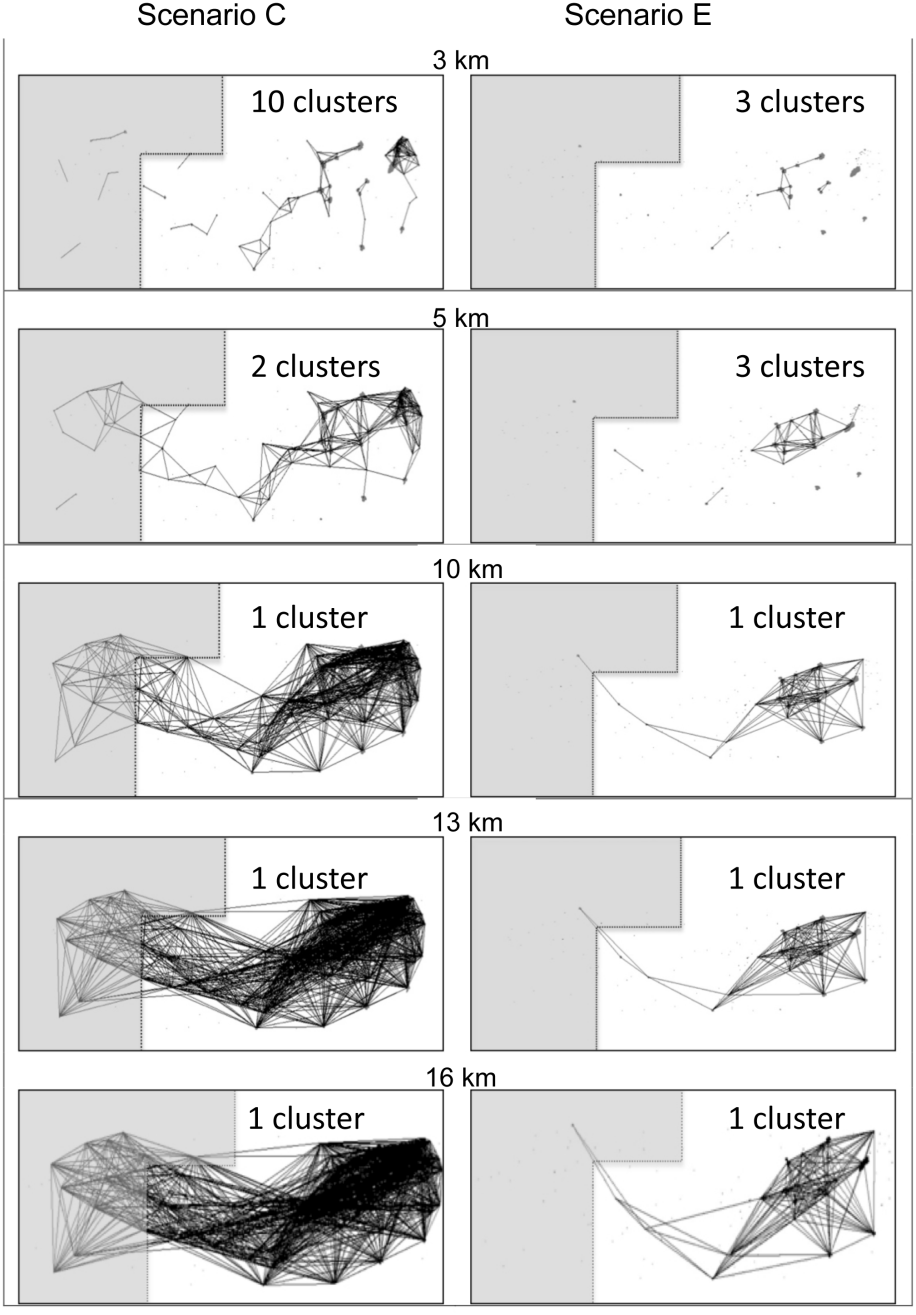
Representations of the network graphs showing the changes in network structure for Scenario C (removal of waterholes ≤2500 m^2^) and E (removal of waterholes ≤10000 m^2^) at 3, 5, 10, 13 and 16 km which represent a range of travel distances for our study species: tapirs (3–13 km), white-lipped peccaries (3–16 km), and jaguars (3–10 km). The two scenarios presented show abrupt changes in connectivity.

The initial waterhole network (scenario A) included 187 waterholes that represented a total area covered by water of 3.14 km^2^ and included 17,391 potential links (when no threshold distance was considered; Fig. 2). For all travel distances, link density abruptly decreased when all small waterholes (2,500 m^2^) were deleted showing a threshold type response at an early stage of the model. The frequency of waterholes for each size class declines as a power function with many small waterholes (n = 115 of ≤2,500 m^2^) and a few large waterholes (n = 15 of ≥40,000 m^2^; Fig. S1). Our base scenario consisted of one connected network for all travel distances, except for the 3 km distance. This suggests that only when individuals move more than 3 km they will be able to reach the remaining waterholes in each scenario. As travel distance decreases, the number of clusters (2 or more linked waterholes) increases (Fig. 3).

### Waterhole Network Structure

Drought dramatically influenced waterhole network structure and therefore the potential connectivity of waterholes and potential suitable habitat for our model species. An abrupt decrease in link density showing a threshold was observed when small waterholes (≤2,500 m^2^) were removed, followed by stabilization in link density curve after scenario E, i.e., when medium size waterholes were removed (10000 m^2^), with similar patterns for the different travel distances considered (Fig. 2). For example, for a travel distance of 13 km (e.g., within tapir and peccaries daily travel capacities), the number of links in the base scenario (scenario A-that included all waterholes larger than 700 m^2^) was 7,424 (42.7% of all potential links, i.e., without a threshold distance). For this same example, scenario C would correspond to a rapid decrease in link density with only 209 links left, which corresponds to 16.3% of the links present in the base scenario. In scenario F (when the link density curve stabilizes) we observed only 94 links left, i.e., 1.3% of all links found in the base scenario.

The removal of waterholes ≤2,500 m^2^ (scenario C), which represents the most frequent waterhole size in our study area, resulted in a loss of 61.5% of the total number of waterholes included in the network (Fig. 2). Most of the waterholes of this size dry fast during the early dry season and are not a reliable source of water. The removal of 90.9% of the waterholes (scenario F) caused a loss of only 15% of the total area covered by water; however, this caused a drastic reduction of more than 95% of the links for all travel distances (Fig. 2, scenario F). For scenario C, network connectivity was maintained inside and outside the reserve when a minimum of 5 km travel distance was considered- a distance within all species movement range (Fig. 3); however, these waterholes were typically observed to dry every year in the field.

A travel distance of 5 km (within all species travel capacities) did not ensure connectivity for all drought scenarios; for example, the sub-network observed inside the reserve had a sparser density of waterholes linked compared to the sub-network outside the reserve, and its connectivity was maintained only when waterholes smaller than or equal to 10,000 m^2^ persisted in the landscape (Fig. S2). In a conservative scenario of waterhole deletion (scenario E) and for a travel distance of 13 km (maximal reported travel distance by tapirs), the sub-network within the reserve collapsed, leaving only three waterholes within the boundary of the reserve linked to the sparse network outside the reserve (Fig. 3). In the most severe drought scenario (K) waterholes became very sparsely connected outside the reserve and non-existent inside the reserve.

### Probability of Connectivity and Node Importance

**Figure 4:**
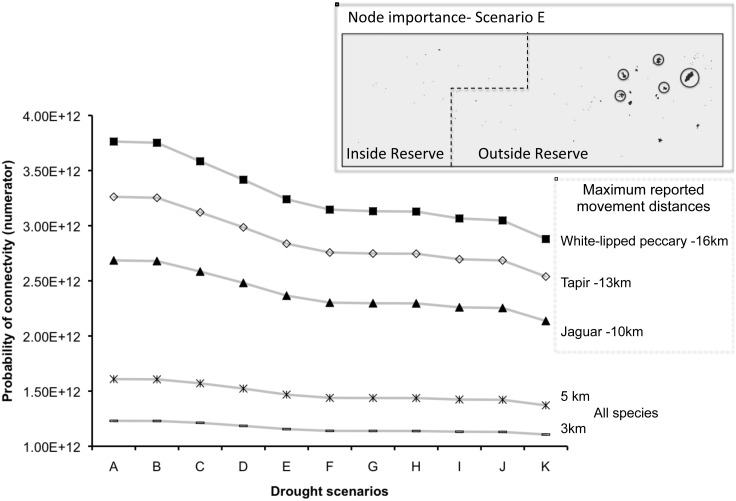
Changes in the probability of connectivity with different travel distances. Inset node importance map illustrates the five waterholes (encircled) with higher ranking based on their contribution to maintaining the overall probability of connectivity of the waterhole network in scenario E for all species (added probability of connectivity of each waterhole by all 5 distance thresholds used).

We observed a decrease in the probability of connectivity as waterholes were removed from the network for each travel distance. The probability of connectivity increased with increasing travel distance. The probability of connectivity for all scenarios was lower at the lower range of travelled distances of all three species (3 and 5 km; Fig. 4). However, we found that even though node importance (given by the probability of connectivity of each node) decreased with waterhole area (smaller waterholes were less important than large waterholes), the location of waterholes further influenced their node importance; therefore, large waterholes located far from other waterholes had lower node importance than smaller waterholes located close to other waterholes. For example, a waterhole of 172,500 m^2^ was ranked 9^th^ with respect to its node importance even though it was larger than waterholes ranked 8^th^ given its location (Fig. S3).

### Suitable Habitat

The waterhole-associated area, or “suitable habitat” using a buffer radius of 1 km around waterholes, corresponded to 52% of the total area in the base scenario. The removal of waterholes ≤10,000 m^2^ (scenario E) caused a large reduction with only 21% of initial suitable habitat remaining (waterhole-associated area in the base scenario; Fig. S4). If we set our buffer radius to 2 km, the removal of waterholes ≤10,000 m^2^ still caused a large reduction of suitable habitat with only 29% of the suitable area remaining. Of the total area covered by our network, only 9% was found within a radius of 1 km and 25% within a radius of 2 km from the existing waterholes in scenario F (simulating the peak of the dry season). Given a buffer radius of 1 km, we found that the number of connected patches of suitable habitat (components of buffered patches) decreased by half (from 13 to 6 connected patches) from scenario E to K. The same pattern was observed when considering buffers of 2 km; only three connected patches of suitable habitat remained in our last scenario, K (Fig. S4).

## Discussion

Our results emphasize that: 1) waterhole number is seasonal and very sensitive to dry seasons, 2) changes in waterhole availability may not sustain a functionally connected network of waterholes for our endangered study species under present and future drought scenarios, and 3) network analysis can improve our understanding of reserve functioning and potential habitat connectivity in highly seasonal landscapes. Our analysis revealed that the potential connectivity of the waterhole network is very sensitive to drought for jaguars, white-lipped peccaries and Baird’s tapirs. By using a range of reported travelled distances, we were able to model the effects of both daily movement patterns around waterholes (suitable habitat analysis) and long, rare movements (connectivity analysis). Our analyses highlight the potential negative effects for three endangered species of observed trends of decreasing precipitation and future projections of changes in drought conditions in the area. The availability of water outside the protected area might result more attractive for water-dependant species, demonstrating the need for further species conservation programs in such a human dominated landscape.

### Loss of Connectivity

Based on our models, maintaining waterholes smaller than 10,000 m^2^ (scenarios A–D) is especially important for the potential connectivity of the landscape both inside and adjacent to the reserve. The distribution and abundance of these small waterholes ensure accessibility to water without large and costly movements. In addition, animals might use these small waterholes as stepping-stones en route to large waterholes, especially to move between sub-networks inside and outside the reserve (Fig. 3). Removing small waterholes caused a non-linear decrease in the connectivity of the network showing that link density abruptly decreases after a threshold of waterhole size removal. When only large waterholes remain in the landscape these are connected by few links (Fig. 3), showing a fragile network of waterholes available for species. Our results can be explained by percolation theory, which suggests that when random habitat loss occurs across more than 60% of the landscape, the largest habitat patch size decreases abruptly and no longer spans the landscape [41]. In our landscape, more than 60% of the waterholes are lost at an early stage of our deletion model (scenario C), which causes an abrupt decrease in link density and connectivity, with only a few large and clumped waterholes remaining.

The results from our network analyses do not imply that tapirs, white-lipped peccaries and jaguars move between waterholes on a daily basis but suggest that movement may be constrained when the species are forced to move to new waterholes due to seasonal or permanent waterhole drying (e.g., due to climate change) or to disturbance (e.g., logging). In addition, we do not expect species to walk in straight lines, but straight-line movements are general considerations relevant to all network connectivity analysis and represent the shortest distance our study species could cover to move between waterholes; therefore, our results showing changes in potential connectivity using maximal distances should be seen as representing the upper limit of potential connectivity. Additional information on species’ water requirements would improve the assessment of the distances species move between waterholes; however this information is not available for these species. Our approach can be applied to test the potential functional resource connectivity of any other temporally and spatially dynamic resource [11], [21], [42], [43].

The potential connectivity of waterholes in the area was severely reduced for our focal species, despite their body size and dispersal capabilities, given drought scenarios that represent the peak of the dry season in the driest years during the study period. Even though a small portion of the network remained connected (Fig. 3), this remnant network fell outside the reserve. Our study emphasizes the importance of considering the spatiotemporal dynamics of resources inside and around protected areas [13], [44]. Even though our study only corresponds to a portion of the Greater Calakmul Region, our fieldwork suggests that these patterns may be common throughout the region.

### Habitat Area vs. Quality?

Our results further emphasize that the spatio-temporal distribution of resources will likely determine the functional connectivity of the landscape. If resources become increasingly rare and then isolated, the chance that they fall outside the movement range of species will increase [45], [46]. In this study, matrix habitat between waterholes is a homogenous-forested landscape, which allows free movement between waterholes. Our movement scenarios were not sensitive to estimates of the resistance to movement of the forested matrix, as is often the case in least-cost connectivity analyses in heterogeneous landscapes. Suitable habitat for the study species considers waterhole use; therefore habitat quality decreases as waterholes dry up causing species to perform longer and unusual movements. In our study, the changes in resource (waterhole) network connectivity in different drought scenarios show dynamic connectivity that will likely not be identified if only habitat area is considered in connectivity analyses; a result that suggests the importance of evaluating habitat quality in addition to habitat extent [11], [47], [48].

Tapirs, white-lipped peccaries, and jaguars possibly require more access to water when temperatures rise during the dry season, either to lower body temperature or, in the case of tapirs and white-lipped peccaries to compensate for the lower water content of their food items [28]. This shows that in very dry scenarios species are likely to be forced to move beyond their usual daily travel distances and in some cases even the maximum reported movement capacities of our model species might be insufficient for individuals to reach water resources. If species regularly perform short distance movements (no more than 2 km) suitable habitat may be seasonally limited in this continuous forested area. In addition, the number of connected patches of suitable area (overlapping buffer areas) decreases suggesting limited movement between suitable areas.

Even if our three model species are able to perform such long distance movements in less than three days [17], [33], [34], these movements are costly and performed rarely. Given the species’ requirements, forested areas with only small waterholes, irrespective of the quality of the forest itself, are thus of decreasing quality as the dry season progresses. As observed in Figure 4, only a small percentage of suitable habitat remains during the most severe drought scenarios we studied. Such a situation was actually observed by Reyna-Hurtado *et al.* [17] in the Calakmul Biosphere Reserve. The four groups of white-lipped peccary they studied behaved like central-place foragers around, and foraged close to, the only remaining waterhole in their 240 km^2^ study area. In addition, the loss of waterholes will not only affect jaguar habitat directly but it will indirectly influence prey availability near waterholes, which will have an overall effect on habitat quality. Therefore, habitat quality must be assessed in terms of resource availability for each species. More generally, although large reserves often contain critical areas of high quality habitat, this may not always be the case and analyses of the sort reported here can inform reserve design and management [49].

### Reserve and Corridor Design and Management

The effect of drought was not uniform across the study region and was particularly apparent within the Calakmul Biosphere Reserve. Climate models predict an increase in temperature and a reduction in precipitation for the area [8]; therefore our study, based on patterns of waterhole drying observed in the field between 2006 and 2009, assumed a realistic drought scenario. Our study allowed us to show that the potential connectivity of the waterhole network is dependent on movement capabilities and is dynamic in space and time. Additionally, only one waterhole keeps the reserve connected to the network outside the reserve (Fig. 1), the loss of which would interrupt a potential resource corridor for individuals moving east to the waterhole network beyond the reserve boundary. The loss of waterholes of this size was observed in the field at the peak of the dry season during a very dry season (e.g., 2006).

Our study region is an important part of the Mesoamerican Biological Corridor and is of considerable conservation value because of its high diversity and area [50]. The Greater Calakmul Region contains large waterholes that ensure water availability throughout the dry season even in years of very low precipitation (e.g. 2005; personal observation). Our study area is represented by a homogenous-forested matrix, which allows free access to waterholes for most species. However, the network analysis revealed higher node importance of large waterholes and documents that the spatial distribution of these large waterholes was heterogeneous and aggregated beyond the boundary of the reserve (Fig. 3). Available habitat outside the reserve might represent a better habitat for species in terms of water resources. Habitat outside the protected area experiences threats caused by human activities such as hunting or habitat disruption by logging and agriculture which can hinder the survival of species. Waterholes located outside the reserve, and with a larger area, showed higher node importances in all scenarios, which emphasize their contribution to maintaining connectivity of the remaining network (Fig. 4). These large waterholes are all located outside the reserve are found close to each other, which suggests that this area might become a refuge for species if drought conditions continue as predicted.

Our results suggest that resource connectivity should be at the centre of reserve network design [51]. This will be especially important if trends of climate or land use change directly impact resource availability. In addition, human populations in the area also depend on water bodies. Critical waterholes important for humans and fauna in the region currently lack a sustainable management plan. The approach we used in this paper can be used to rapidly assess landscape viability and vulnerability for a range of species specifically considering variations across space and time. Our approach can also be used to initially prioritize habitat and resources for landscape management programs and to target further data collection and monitoring [47], [49] in areas where critical resources are spatially and temporally dynamic. Our results emphasize the need to better understand the availability of water inside the reserve and the consequences for species survival in this protected area. Conservation actions are needed outside the reserve not only to ensure the survival of species in areas with low waterhole abundance, but also to identify areas of potential human-animal conflicts if animals move outside the reserve to find water (or other resources). A higher rate of hunting and crop-raid events might be expected outside the reserve; therefore, further studies on these topics are needed to inform conservation actions.

Given the rapid effects of climate change, which in some areas has now translated into altered precipitation regimes [21], we require new approaches to create dynamic reserve and corridor network designs that incorporate the temporal and spatial dynamics of resources [43], [51]. This study considered the effect of climate change on species persistence by evaluating the effect of changes in precipitation in water resource availability and the connectivity of resources. We have provided science-based information that can inform future conservation programs in the area. These programs can be established for the protection of key water bodies inside and outside the reserve, for the conservation of areas where water bodies with higher node importance value were found, and to promote further studies on the movement capacities of these endangered species, their water requirements, and the potential consequences of a higher abundance of these species outside the protected area.

### Future Considerations

Our analysis suggests that even though waterholes may remain connected during the wet season, resource connectivity is abruptly affected during the dry season. If current trends of precipitation continue, drought periods are expected to be longer and affect the waterhole network. In particular, our results suggest that the loss of a small number of water holes has a large effect on the network’s structure and connectivity. These effects will increase in strength when dry years occur consecutively and may force species to move to unprotected areas beyond the reserve. Currently this effect is seasonal but it may reflect future scenarios with longer and more severe dry seasons. A permanent shift in conditions would have consequences for species persistence in and around the reserve. Climate and land use changes will dramatically alter the functional connectivity of this region, and hence the conservation capacity of the Calakmul Biosphere Reserve.

Our network approach allowed us to link field and GIS data to analyse the potential connectivity of a waterhole network for three large mammal species of conservation concern. Although this modelling approach has been applied to study the impacts of habitat fragmentation [4], [19] few results are available for water resource networks [52] and their dynamics [53]. We recognize that detailed data and habitat use patterns are still missing from our model. However, our modelling approach is easily updated as movement data (e.g., from GPS collars) becomes available. Next steps will involve modelling demography to allow the identification of key features of the network that are critical for metapopulation persistence under climate change [11], [20]. Furthermore, changing human land use in the area might influence species’ movements in the future, so the inclusion of land use change (e.g. logging roads or human settlement) will also be important to evaluate change in landscape connectivity.

## Supporting Information

Figure S1 **Power relationship between waterhole size and frequency, R^2^ = 0.64.** Each dot corresponds to a drought scenario described in the text. Note the log scale of both axes.
(TIFF)

Figure S2 **Network graphs showing changes in network structure when considering a 5 km travel distance for scenarios A (all waterholes considered), C (waterholes ≤2500 m^2^ removed), E (waterholes ≤10000 m^2^ removed), and J (waterholes ≤50600 m^2^ removed).** The grey area corresponds to the reserve.
(TIF)

Figure S3 **Area of patches with the ten highest node importance values.** The symbols correspond to each of the 11 drought scenarios A to K.
(TIF)

Figure S4 **Percentage of suitable habitat lost in each drought scenario.** The upper figures show the buffer analysis graphs with waterholes remaining in drought scenario E and K and their 1 km and 2 km buffers.
(TIF)

File S1 **Imagery details.**
(DOCX)
