# Supplementary figures and images for: The Potential Connectivity of Waterhole Networks and the Effectiveness of a Protected Area under Various Drought Scenarios
Source: PLoS One. 2014 May 15;9(5):e95049. doi: 10.1371/journal.pone.0095049 (PMC4022619; doi:10.1371/journal.pone.0095049)

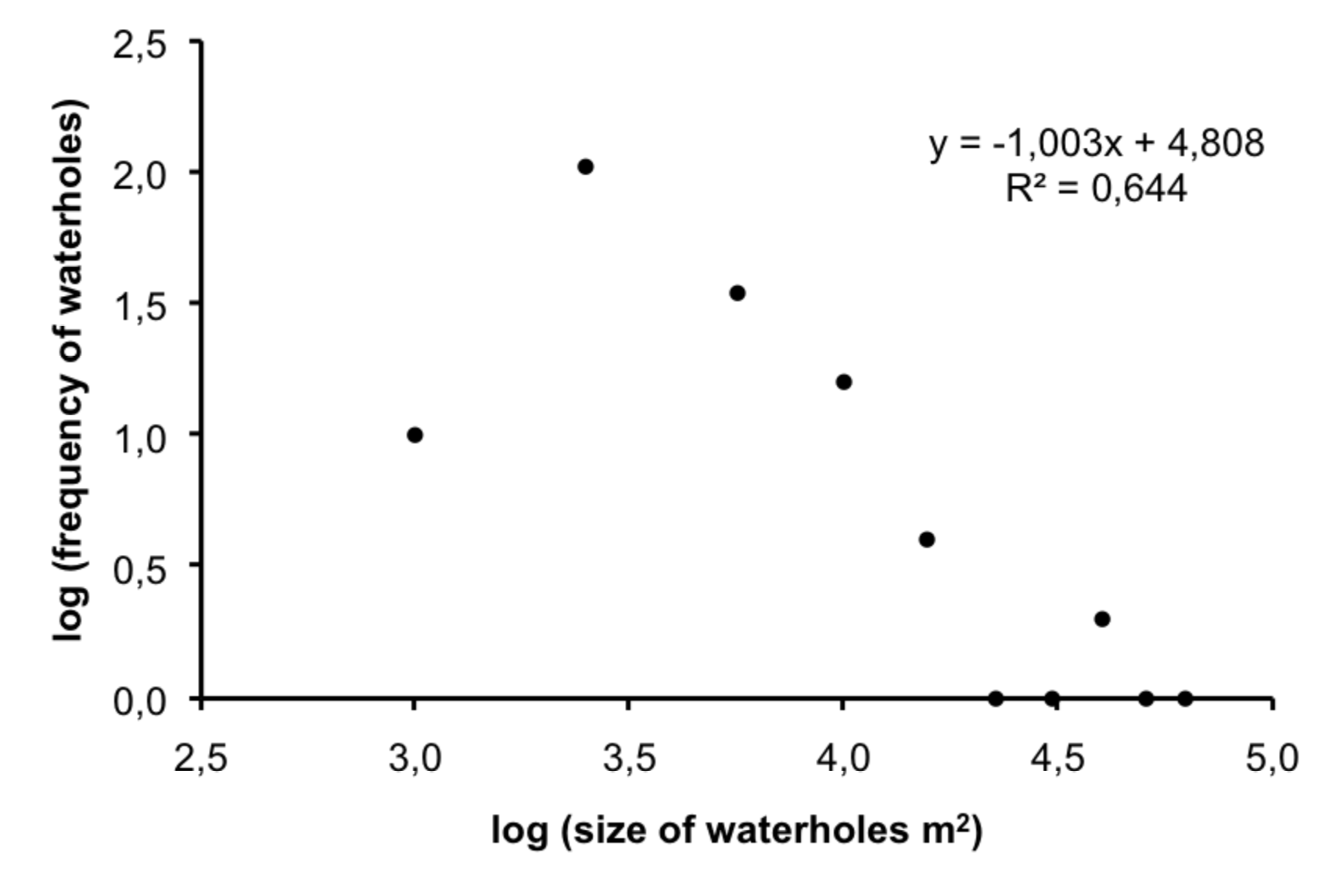

Supplement: Figure S1 — Power relationship between waterhole size and frequency, R2 = 0.64. Each dot corresponds to a drought scenario described in the text. Note the log scale of both axes. (TIFF) [file pone.0095049.s001.tiff]

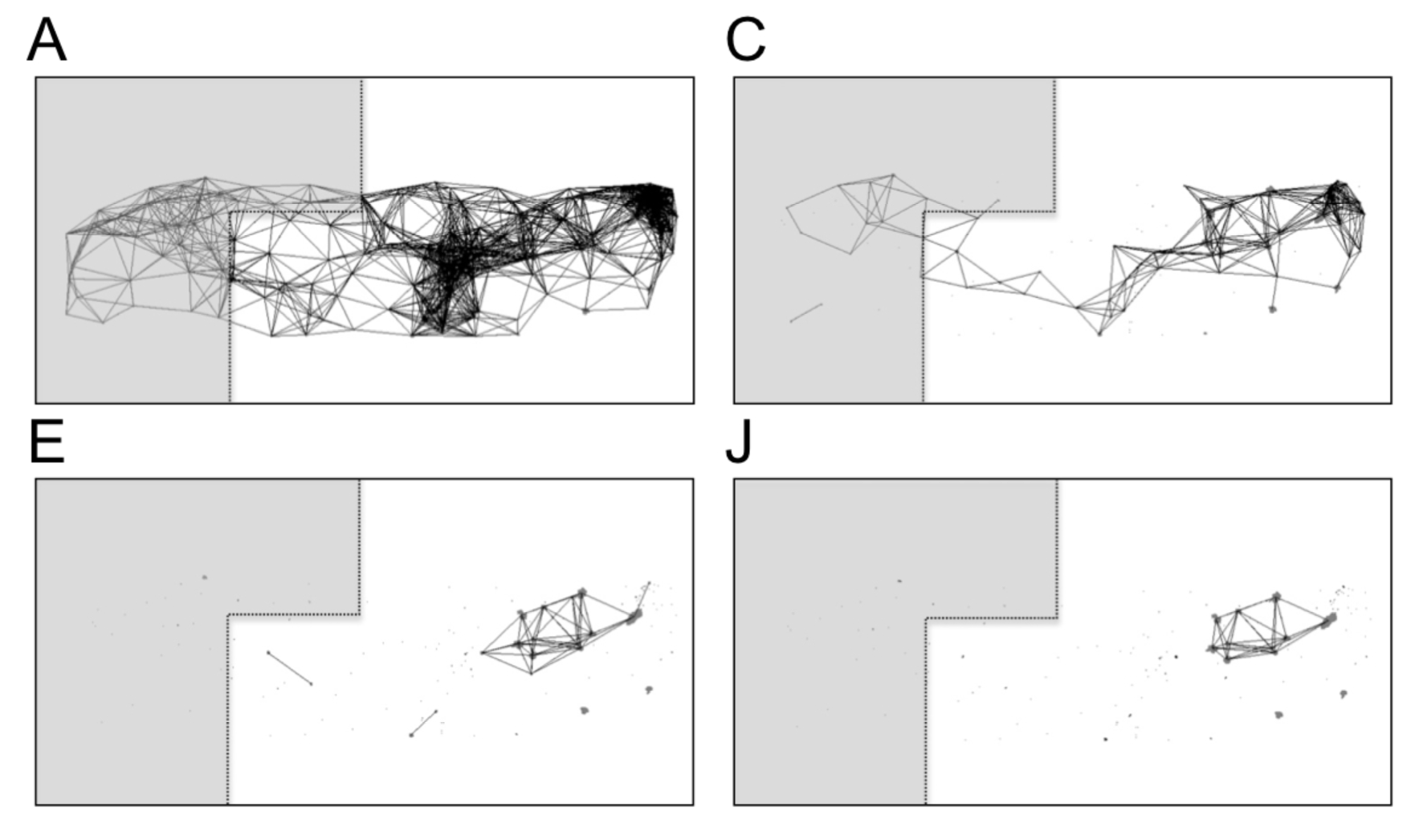

Supplement: Figure S2 — Network graphs showing changes in network structure when considering a 5 km travel distance for scenarios A (all waterholes considered), C (waterholes ≤2500 m2 removed), E (waterholes ≤10000 m2 removed), and J (waterholes ≤50600 m2 removed). The grey area corresponds to the reserve. (TIF) [file pone.0095049.s002.tif]

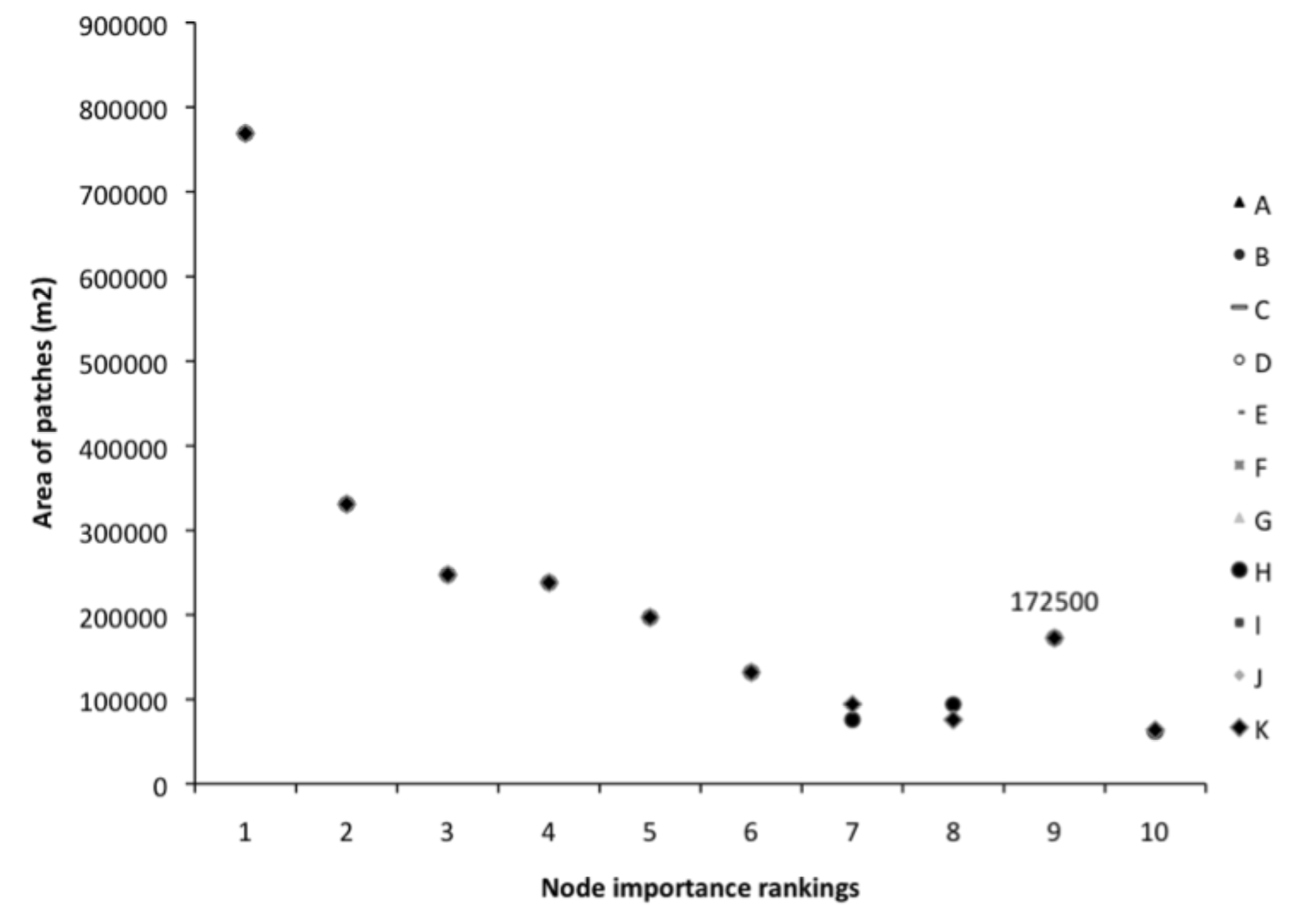

Supplement: Figure S3 — Area of patches with the ten highest node importance values. The symbols correspond to each of the 11 drought scenarios A to K. (TIF) [file pone.0095049.s003.tif]

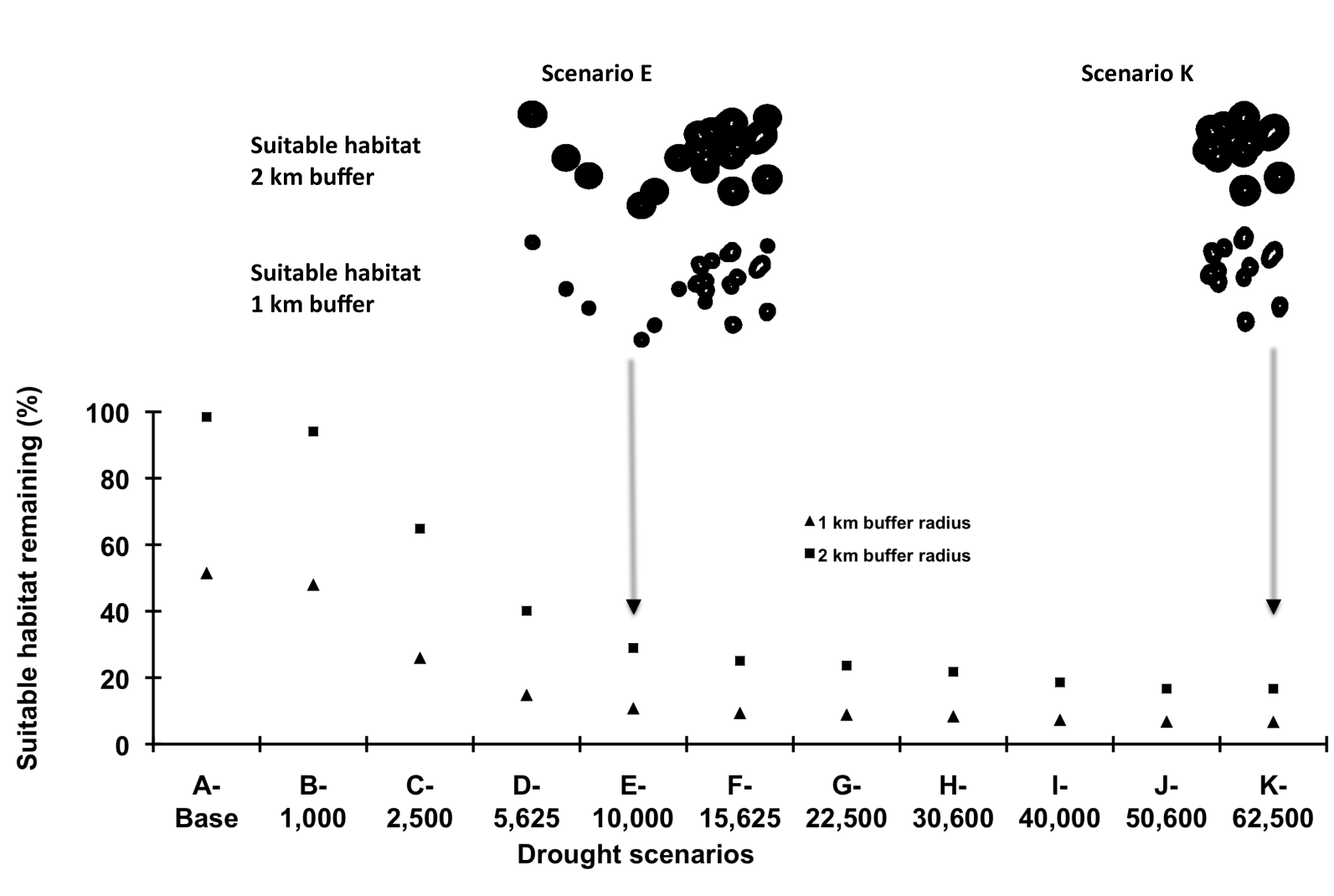

Supplement: Figure S4 — Percentage of suitable habitat lost in each drought scenario. The upper figures show the buffer analysis graphs with waterholes remaining in drought scenario E and K and their 1 km and 2 km buffers. (TIF) [file pone.0095049.s004.tif]
