# Supplementary material for: The Potential Connectivity of Waterhole Networks and the Effectiveness of a Protected Area under Various Drought Scenarios
Source: PLoS One. 2014 May 15;9(5):e95049. doi: 10.1371/journal.pone.0095049 (PMC4022619; doi:10.1371/journal.pone.0095049)
Supplement: File S1 — Imagery details. (DOCX) [file pone.0095049.s005.docx]

**Supporting Information**

*Imagery details*

We used visual interpretation and on-screen digitization techniques to create polygons representing waterhole boundaries based on appearance (form and texture) and edge patterns. As expected, visual inspection of orthorectified aerial photographs with higher spatial resolution provided better digitizing accuracy than NDVI data layer obtained from the Landsat ETM+ image. However, while the finer resolution of the aerial photographs allowed greater spatial accuracy, the absence multiple spectral bands made it difficult to differentiate small waterholes from tree shadows or topographic features.
